# Supplementary material for: Americans experience a false social reality by underestimating popular climate policy support by nearly half
Source: Nat Commun. 2022 Aug 23;13:4779. doi: 10.1038/s41467-022-32412-y (PMC9399177; doi:10.1038/s41467-022-32412-y)
Supplement: Supplementary file 1 — Supplementary Information File [file 41467_2022_32412_MOESM1_ESM.pdf]

Supplemental Material

Americans Experience a False Social Reality by Underestimating Popular Climate Policy Support by Nearly Half

Gregg Sparkman[1]*, Nathan Geiger[2], and Elke U. Weber[3]

Author Affiliations:
[1]Department of Psychology and Neuroscience, Boston College, Chestnut Hill, MA 02467
[2]Media School, Indiana University Bloomington, Bloomington, IN 47405
[3]Andlinger Center for Energy and Environment, Princeton University, Princeton, NJ 08540

Figure S1.
Perceived State Climate Change Worry and Support for Climate Policies VS Actual State Levels

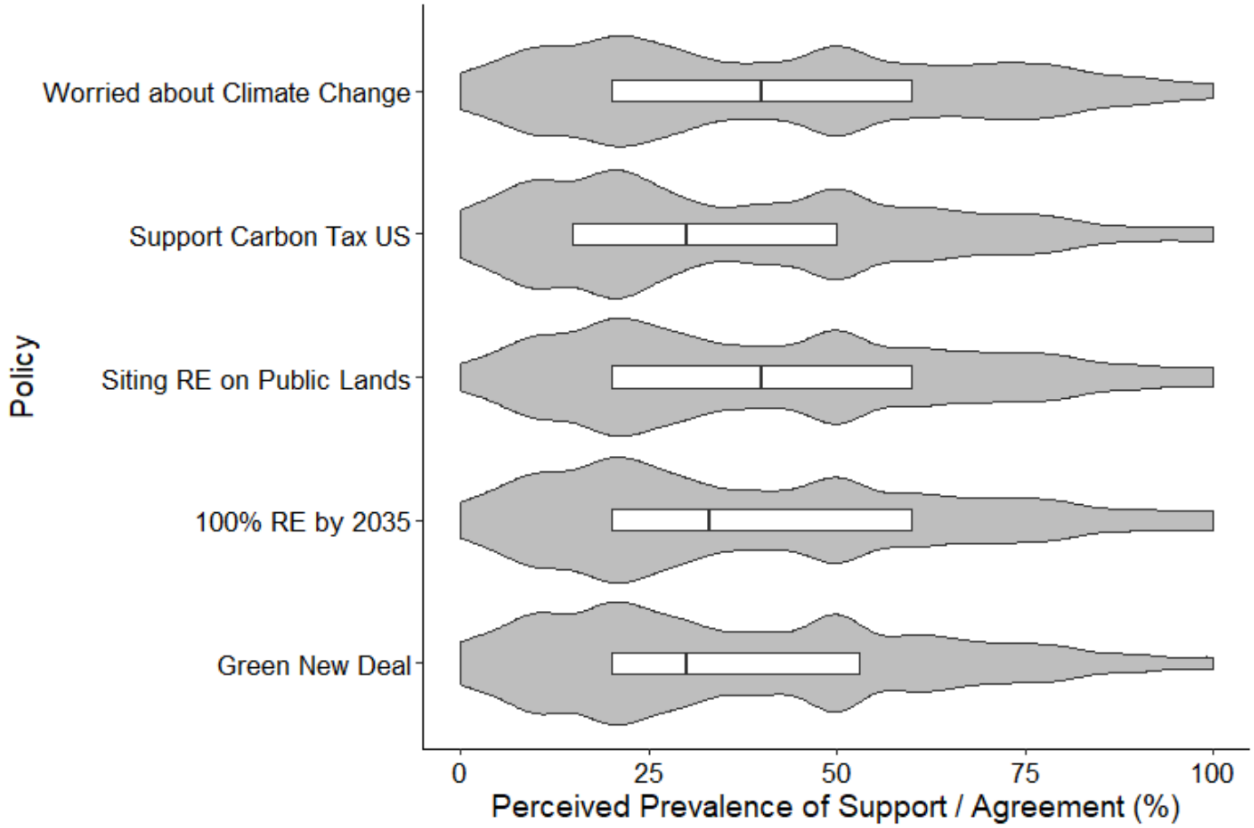

Note. Boxes inside the violin plot represent the middle 50% of the sample, with a line at the median, while the minima and maxima illustrated represent the full range of responses (from 0% to 100%). Results shown here are weighted to be nationally representative, and are averaged across all state-level estimates. RE refers to renewable energy. *N*=6119 survey participants.

Table S1. Differences in Real vs Perceived National Policy Support

Policy	Actual Support (%)	Perceived Support (%)	*T (df)*	Cohen's *d*	*p*
Carbon Tax	67	36.6	27.27 (1410)	1.21	< 0.001
Siting RE	80	43.4	44.01 (1429)	1.45	< 0.001
100% RE	66	39.5	31.92 (1432)	1.05	< 0.001
GND	66	37.9	33.96 (1403)	1.11	< 0.001

Note. Real, perceived and 95% CI are all in percentages. Welch two sample t-tests were conducted using observed data for norm perceptions and the YPCCC polls and their reported margin of error for the of real policy support values. RE refers to renewable energy. GND refers to the Green New Deal.

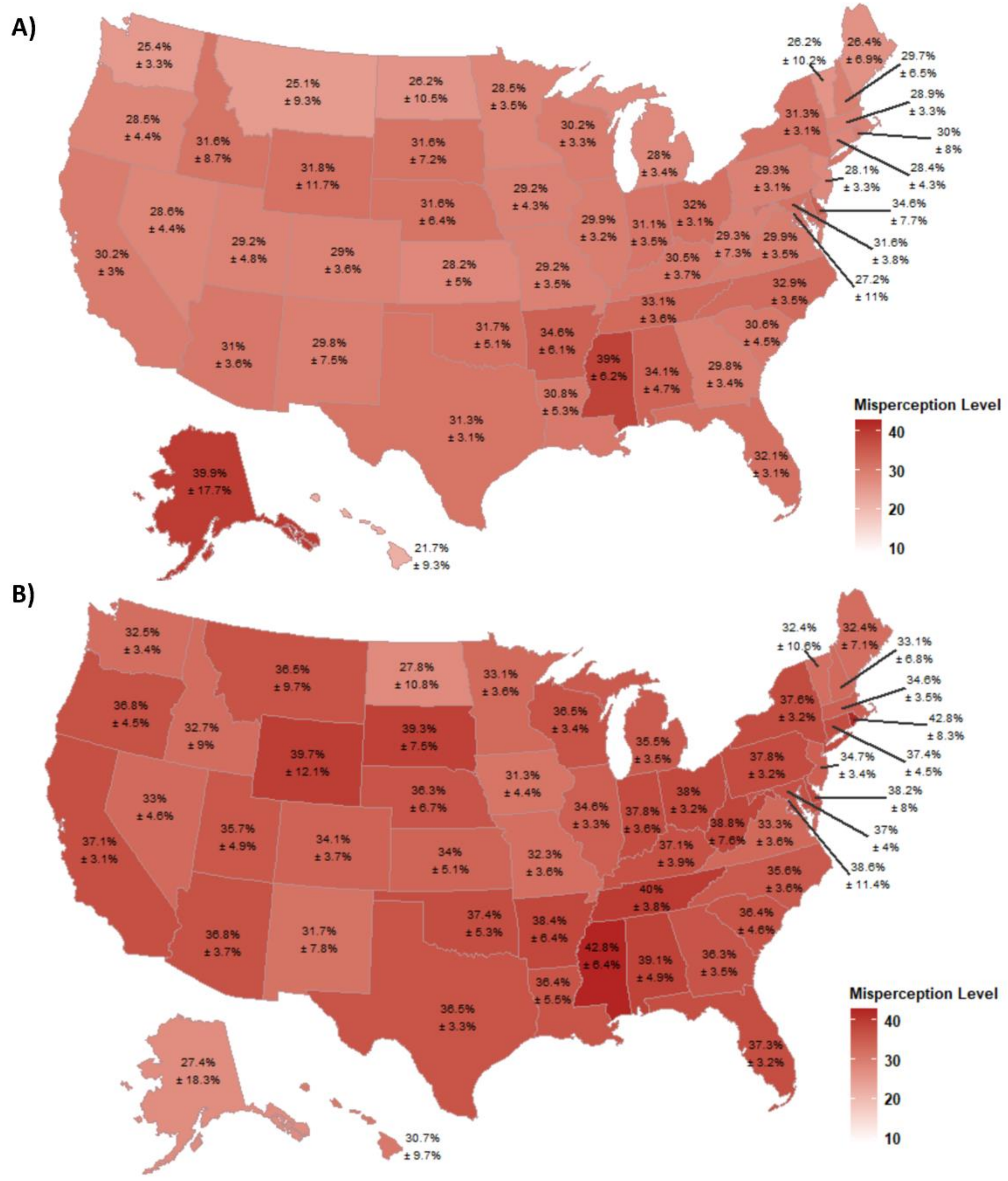

Fig. S2. Panel A) shows pluralistic ignorance levels for support for a carbon tax. Panel B) shows levels of pluralistic ignorance for siting renewables on public lands. Both are calculated by taking the difference from real and perceived national levels of support and averaging those levels across participants in each state (greater values indicate real levels are higher than perceived norms, i.e. greater underestimation in perception; N=6119). The ± values represent the margin of error (half of the 95% confidence interval calculated for each state's mean).

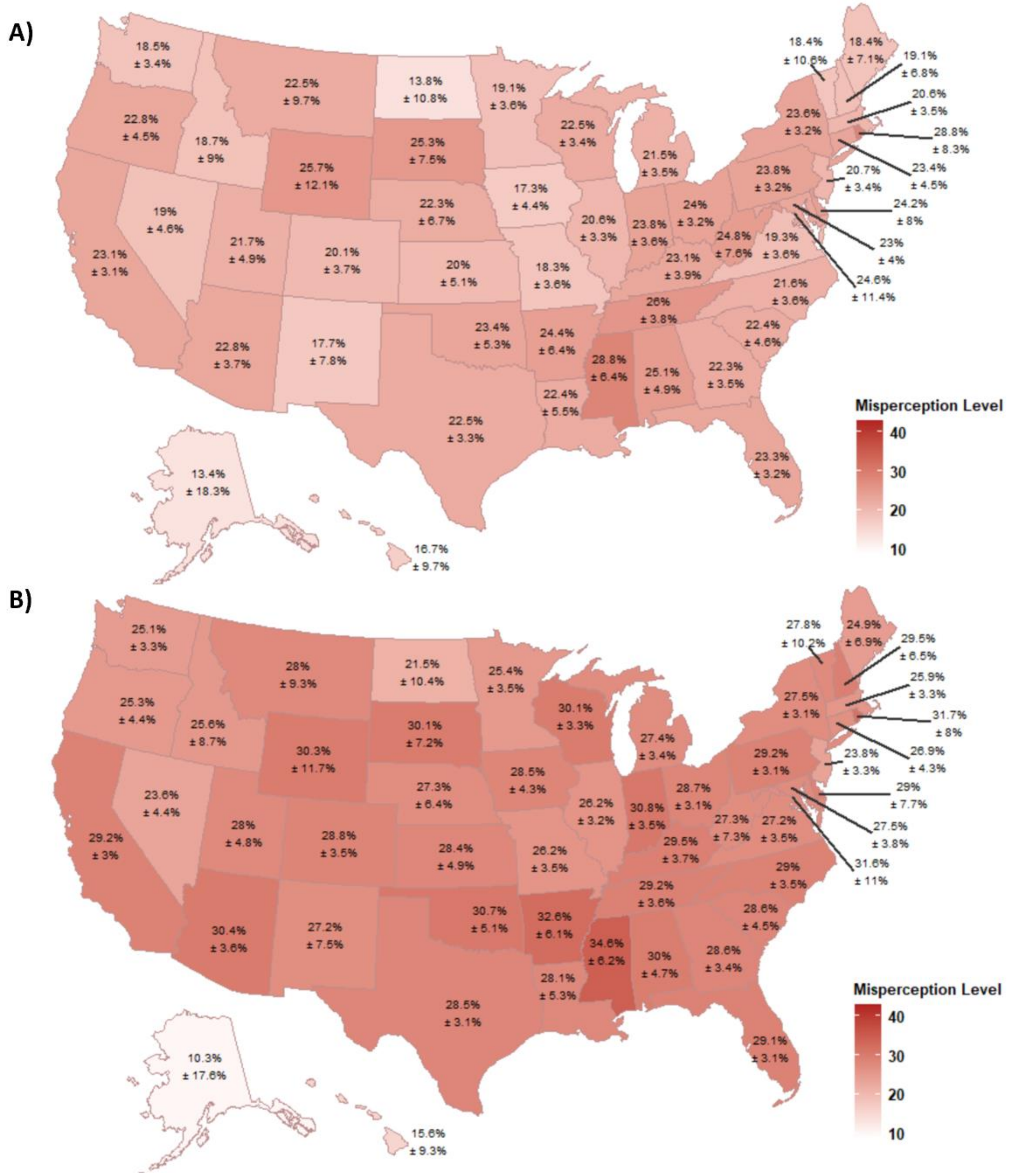

Fig. S3. Panel A) shows pluralistic ignorance levels for support for a 100% renewable energy mandate. Panel B) shows levels of pluralistic ignorance for the Green New Deal. Both are calculated by taking the difference from real and perceived national levels of support and averaging those levels across participants in each state (greater values indicate real levels are higher than perceived norms, i.e. greater underestimation in perception; N=6119). The ± values represent the margin of error (half of the 95% confidence interval calculated for each state's mean).

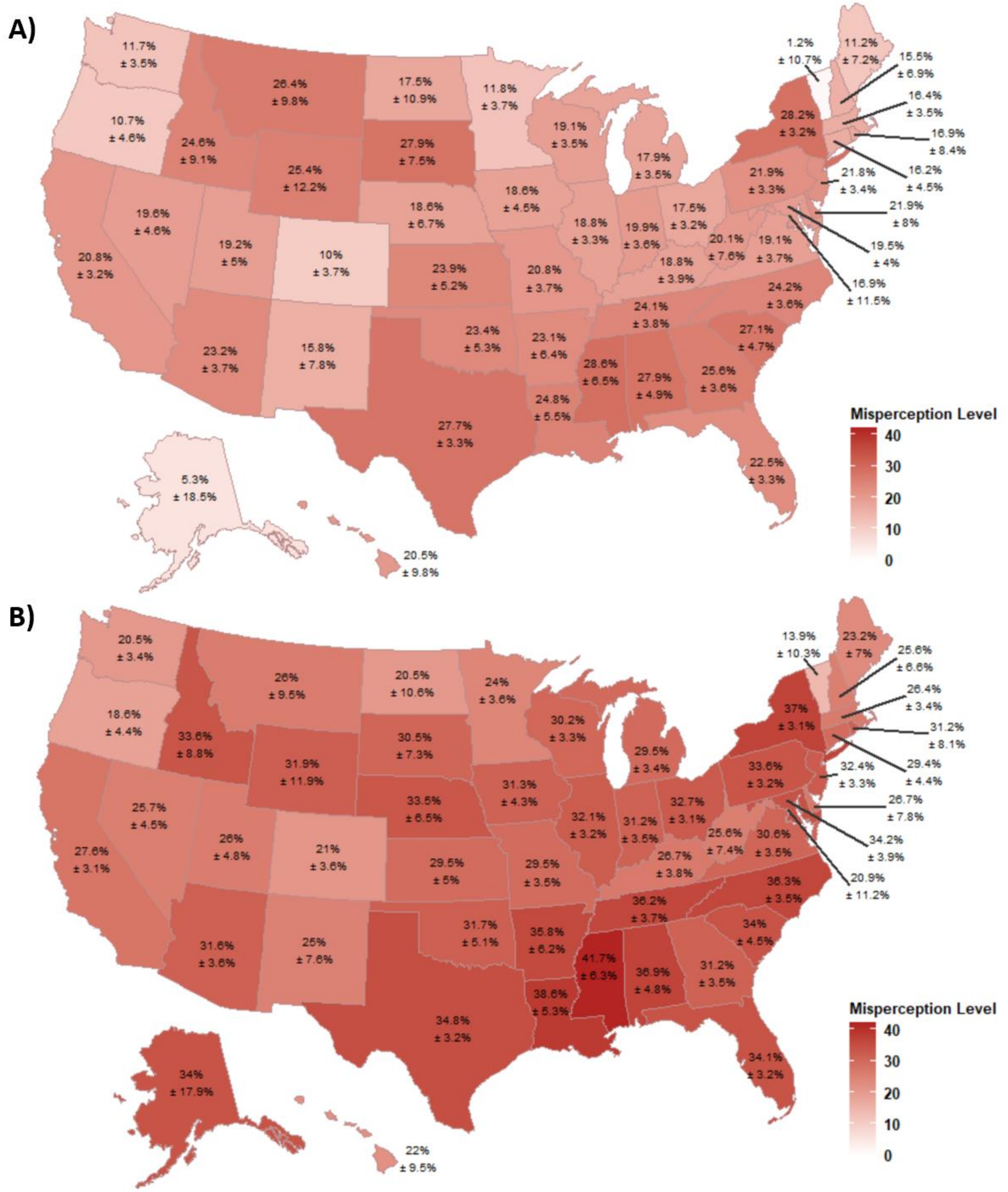

Fig. S4. Panel A) shows pluralistic ignorance levels for state-level estimates for worry about climate change. Panel B) shows levels of pluralistic ignorance state-level estimates of support for a carbon tax. Both are calculated by taking the difference from real and perceived state levels of concern (for A) or support (for B) and averaging those levels across participants in each state

(greater values indicate real levels are higher than perceived norms, i.e. greater underestimation in perception; N=6119). The ± values represent the margin of error (half of the 95% confidence interval calculated for each state's mean).

Table S2. Pluralistic Ignorance Levels by Demographics, Results from Multiple Regression

Variable	F (df)	P	Low Level [95% CI]	High Level [95% CI]
Political Orientation	655.5 (1, 38316)	< 0.001	Very Liberal = 22.1 [17.98, 26.17]	Very Conservative = 33.2 [29.1, 37.30]
Race	46.4 (7, 35116)	< 0.001	White = 26.4 [22.32, 30.48]	Black = 35.3 [31.17, 39.62]
Area Type	51.6 (2, 38196)	< 0.001	Suburban = 25.9 [21.83, 30.00]	Urban = 29.2 [25.04, 33.26]
Income	48.1 (1, 38858)	< 0.001	\$150K-\$200K = 26.2 [21.00, 29.24]	< \$5000 = 29.9 [25.76, 34.06]
Employment	30.8 (8, 36897)	< 0.001	Unemployed = 21.6 [17.24, 25.99]	Full-Time = 30.1 [26.03, 34.22]
Age	23.8 (1, 38141)	< 0.001	20 years old = 25.3 [21.16, 29.47]	100 years old = 30.7 [26.41, 35.01]
Marital Status	9.0 (4, 39107)	< 0.001	Living w/Partner = 24.7 [20.55, 28.92]	Married = 27.7 [23.65, 31.83]
Gender	4.3 (1,38058)	0.039	Female = 27.0 [22.96, 31.13]	Male = 27.7 [23.58, 31.75]
Education	4.1 (1, 38001)	0.042	Doctorate = 26.7 [22.59, 30.85]	No Diploma = 28.0 [24.23, 32.62]
# of Children	2.0 (1, 39011)	0.156	6 Children = 26.0 [21.46, 30.45]	None = 27.4 [23.36, 31.52]

Pluralistic ignorance levels are calculated by taking the difference of perceived values from real values for all worry and policy items with both available (all in percentages), and then regressed on the demographic variables shown here in mixed model multiple regression with random intercepts for participant and item, and weighted to be nationally representative. All significance tests are two-tailed. To illustrate the range of pluralistic ignorance by each demographic feature, a high and low level are shown with estimates and confidence intervals.
