# Supplementary material for: Americans experience a false social reality by underestimating popular climate policy support by nearly half
Source: Nat Commun. 2022 Aug 23;13:4779. doi: 10.1038/s41467-022-32412-y (PMC9399177; doi:10.1038/s41467-022-32412-y)
Supplement: Supplementary file 3 — Description of Additional Supplementary Information [file 41467_2022_32412_MOESM3_ESM.pdf]

Description of Additional Supplementary Information

Americans Experience a False Social Reality by Underestimating Popular Climate Policy Support by Nearly Half

Gregg Sparkman[1]*, Nathan Geiger[2], and Elke U. Weber[3]

Author Affiliations:
[1]Department of Psychology and Neuroscience, Boston College, Chestnut Hill, MA 02467
[2]Media School, Indiana University Bloomington, Bloomington, IN 47405
[3]Andlinger Center for Energy and Environment, Princeton University, Princeton, NJ 08540

Title: Supplementary Data File 1
Description: This data file contains all participant-level data used in this publication.

Title: Supplementary Data File 2
Description: This date file is a codebook for the participant-level data in Supplemental Data File 1.

Title: Supplementary Data File 3
Description: This data file contains all state- or nation-level data used in this publication

Title: Source Data
Description: This date file contains data used to create all figures in this publication.
